# The Soluble Plasminogen *Activator Receptor* as a Biomarker on Monitoring the Therapy Progress of Pulmonary TB-AFB(+) Patients

**DOI:** 10.1155/2010/406346

**Published:** 2010-10-04

**Authors:** Tri Yudani Mardining Raras, Iin Noor Chozin

**Affiliations:** ^1^Laboratory of Biochemistry-Molecular Biology, Faculty of Medicine, Brawijaya University, Malang 65145, Indonesia; ^2^Department of Pulmonology, Faculty of Medicine, Brawijaya University Malang-Dr. Saiful Anwar Hospital Malang, Malang 65145, Indonesia

## Abstract

The role of soluble *soluble urokinase*-type *plasminogen activator receptor* (suPAR) as a biological marker for TB treatment efficacy on active pulmonary TB-AFB(+) patients was investigated. Twenty pulmonary TB-AFB(+) patients participated in a cohort study for six months. The plasma suPAR level was measured using ELISA method before treatment, two months, four months and six months after treatment. At the same time clinical parameters were also measured. Results indicated that all patients (*n* = 20) showed highest plasma suPAR levels before treatment (median 12.775 ng/mL) and significantly decreased ( *P* = .0001<.05, *R* ^2^ = .890) after 2 months (median 8.019 ng/mL) and 4 months (median 5.771 ng/mL) of treatment, respectively. However, only slightly declined after 6 months therapy (median 5.009 ng/mL), near control group level (median 4.772 ng/mL). Interestingly, the significant reduced of suPAR level was parallel to treatment efficacy and correlated with other clinical and laboratory parameters, that is, decreasing of patients' complaints, increasing of BMI (*r* = −0.281), thoracic imaging improvement, sputum conversion, decreasing of ESR (*r* = 0.577) and monocytes count (*r* = 0.536) with exception the width of lesion in thoracic imaging. In conclusion, the suPAR level in could reflect the progress of TB therapy.

## 1. Background

The success in controlling *Tuberculosis* (TB) depends to a great extent on the appropriate diagnosis, the right therapy, treatment monitoring, and evaluation. Particularly concerning the treatment, the long duration of therapy may adversely affect patient adherence to treatment and lead to failure of the recommended treatment program. This urges a stringent monitoring program that could show the progress of the therapy earlier than normal therapy (min. 6 months). In the last decade, several biological markers have been intensively studied. Such markers could contribute, the improvement of the quality of clinical trials; and therefore enable development and validation of new therapeutic strategies. 

One of the most intensively investigated biomarkers is *urokinase plasminogen activator receptor* (uPAR), a cellular receptor for serine protease *urokinase plasminogen activator* (uPA). UPAR can be cleaved from the cell surface by a number of protease, such as chymotrypsin, phospholipases C, and uPA, to yield a soluble form of the receptor (suPAR) that has intrinsic chemotactic properties [1, 2]. One study has demonstrated elevated levels of suPAR in pulmonary TB patient with higher suPAR levels in smear microscopy-positive patients compared to smear-negative patients [3]. SuPAR is an inflammatory biomarker and thus not specific for TB, but it is elevated by several infectious diseases and s to reflect the severity of disease and may hence be used as a prognostic tool. However, little is known about suPAR as a prognostic marker in extrapulmonary TB [4]. 

Assuming that serum levels of suPAR reflect the progress of the therapy, we investigated to access in prospective manner whether serum levels of suPAR may be used to monitor treatment of pulmonary TB-AFB(+) patients. In the present study, the suPAR levels of TB pulmonary patients were measured during-six-month therapy and compared with other routine laboratory evaluation including Body Mass Index (BMI), monocyte count, Erytrocyte Sedimentation Rate, (ESR) and thorax imaging. 

## 2. Material and Methods

### 2.1. Subjects

This study was conducted in Malang, where the prevalence of new smears or cultures positive for TB was 107/100.000 population per year between 2008 and 2009. For the study, 5 tuberculin skin test positive healthy community controls and 20 positive, newly diagnosed pulmonary TB with sputum AFB-(Acid-fast bacillu) positive patients were enrolled. The inclusive criteria were patients with category I of pulmonary TB who regularly took medication, were male and female aged between 15–50 years, had a BMI (Body Mass Index) >16, agreed to be the subject of the research, and signed the informed consent. Exclusion criteria were TB patients with other diseases (severe bacterial pneumonia, HIV-AIDS, and heart disease, diabetes mellitus, heart and kidney problems), extra pulmonary TB, pregnant patients, and patients with psychiatric problems. 

The patients received a six-month (26 weeks) directly observed short course antituberculosis therapy as recommended by the Indonesian National Tuberculosis Program based on WHO TB guidelines.

### 2.2. TB Treatment

The drug regimen consisted of a fixed weight-dependent combination of INH (320–400 mg/day), rifampicin (480–600 mg/day), ethambutol (800–1200 mg/day), and pyrazinamide (1000–1250 mg/day) for the two-month intensive phase, followed by rifampicin and INH for the four-month continuation phase.

### 2.3. Routine Examination

Patient's complaint was conducted prior to the clinical and laboratory examination, body mass index, monocyte count, and Erythrocyte Sedimentation Rate (ESR). All were measured according to the standard procedure conducted in Indonesian Hospital [5]. 

### 2.4. ChestX-Ray Grading of Disease

Standard postero-anterior and lateral chest radiographs were taken prior to therapy and were read by a pulmonologist. The grading included the following categories: (i) minimal lesion, that is, lesion width is less than area restricted to median line, apex and front costae, solitaire lesion can be everywhere, and there is no cavity found, (ii) moderate advanced, that is, width of cavity is less than one lobe, and if there is cavity, should be more than one lobe. (iii) far advance is width of lesion that more than minimal and moderate lesion, if with cavity, should not be more than 4 cm.

### 2.5. Sampling Handling

Study materials were 3 mL blood specimens obtained from new patients through aspiration using injective needle before they started on antituberculosis diagnosis at two, four, and six months after the initiation of anti-TB drugs treatment based on WHO guideline (2HRHZE/4H3R3) [5, 6]. Serum was separated by centrifugation (6000 x g) at 4°C for 7 min and aliquots of 500 mL stored at −80°C.

### 2.6. Enzyme-Linked Immunoassaysorbant Assays

Serum suPAR measurement was done in duplicate using commercially available ELISA kits according to the manufacture's protocol (suPARnostic, ViroGates A/S, Copenhagen, Denmark) [7]. Plate reading was conducted using a Biotech micro plate reader set to 450 nm, with the wavelength correction set to 650 nm. Concentrations of the respective analyses were determined using SPSS version 3.4 software. 

### 2.7. Statistical Analysis

All statistical analysis on suPAR was carried out using SPSS software version and the statistical programming language. Analyzed statistically, using longitudinal model analysis, bivariate analysis, multivariate analysis and linear regression, these analysis were carried out with the SAS 9.13 (SAS Institute, Cary, NC, USA) and SPSS 16 (SPSS Inc, Chicago, IL) program. 

The difference between the patients and the controls was analyzed using one-way ANOVA. The association between the immune parameters and the extent of disease was assessed using the Mann Whitney *U*-test. A *P*-value of *P* ≤ .05 was judged significant. 

### 2.8. Ethics

Written consent was obtained from all participants. The study was approved by the Ethics Committees, Saiful Anwar Public Hospital, Brawijaya University.

## 3. Results

Twenty patients with active pulmonary TB were observed during the six-month treatment. Characteristics of the twenty patients are presented in Table 1. 

The number of female pulmonary TB patients who fulfilled the inclusion and exclusion criteria were (50%) equal to the number of male patients (50%). Distribution based on age in this study showed that TB patients were mainly dominated by those aged between 19–30 years. 

**Figure 1:**
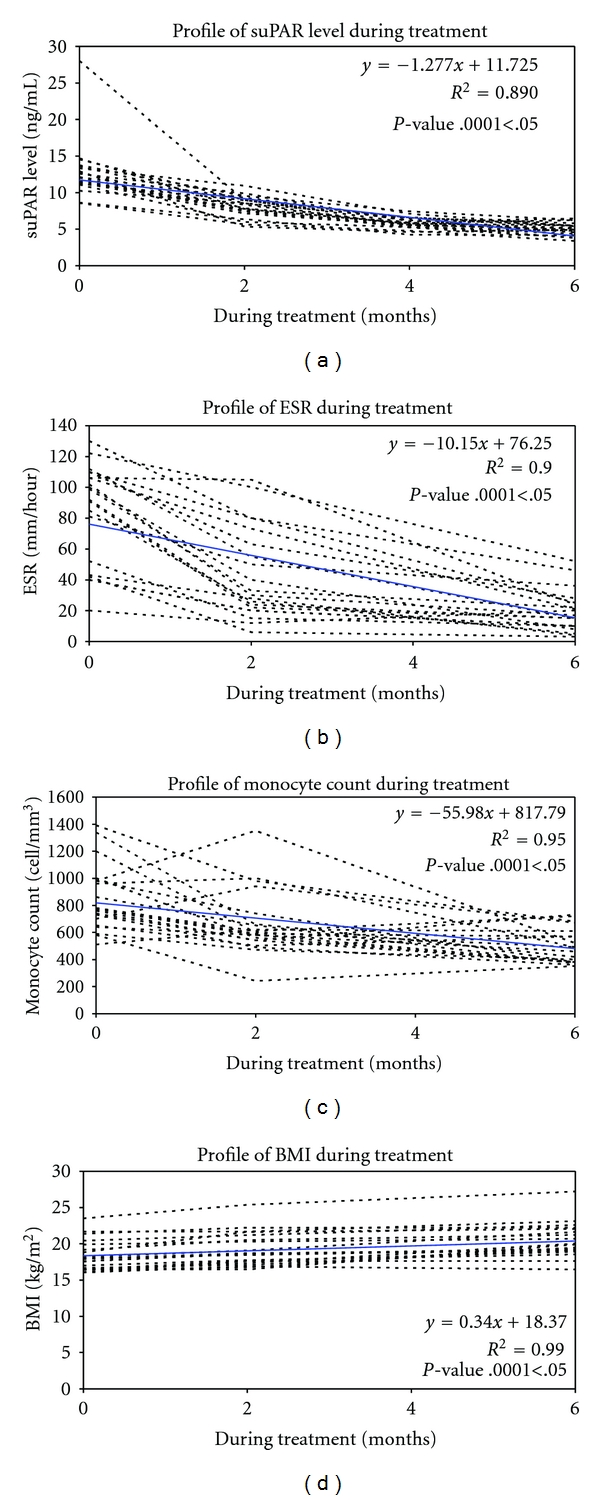
(a) Serum levels of soluble urokinase receptor (suPAR), (b) sedimentation rate of erythrocytes (ESR), (c) monocyte count (d) Body Mass Index (BMI) in 20 TB patient before (0), during (2 and 4 months), and after (6 months) completion of anti-TB treatment. Individual profile (dotted line) and median (thick line) levels are shown.

Measurement of suPAR concentration indicated that there was a high level of suPAR in infected patients before treatment (median 12.775 ng/mL), but suPAR levels dropped significantly (*P* = .0001 < .05) after two months (median 8.019 ng/mL) and four months (median 5.771 ng/mL) of therapy. However, at the sixth month of treatment, there was only slightly decrease although significant reached (median 5.009 ng/mL) (Figure 1(a)). These were almost similar to the healthy control group (median 4.772 ng/mL). 

With regard to clinical symptoms (Table 2), chronic cough is the most common complaint, however, decreasing of appetite, low grade fever, shortness of breath, haemoptoe, and chest pain were also found. After the second and the sixth month of treatment, some patients still complained about coughing, shortness of breath, and chest pain. Concerning sputum conversion, before treatment, all patients (*n* = 20) had sputum-AFB(+) but after the two-month treatment, fifteen patients turned to be AFB(−), and by the end of the six-month treatment, all patients showed sputum conversion. During observation, most patients (19) showed high level ESR, and it decreased significantly (*P*-value .0001 < .05) from time to time during the six-month treatment. ESR usually increased in active processes, but normal ESR did not eliminate the diagnosis of TB [5]. In this papaer, the highest median of ESR before treatment was 84.2 mm/hour, and then it dropped to 44.1 mm/hour after-two-month treatment. The lowest of the ESR level was reached after six-month treatment (19.3 mm/hour). (Figure 1(b)) A significant correlation between suPAR and ESR was found (*P*-value .0001 < .05), with a quite strong correlation coefficient (*r* = 0.577).

The same trend was found on monocytes count. Before treatment, monocytes count showed the highest concentration (median 847 cell/mm^3^) However, it dropped significantly (*P* = .0001 < .05) after two (median 662 cell/mm^3^) and-six-month treatment (median 497 cell/mm^3^) (Figure 1(c)). The decreasing suPAR level correlated significantly (*r* = 0.536) with the decreasing of monocytes count (*P*-value .000 < .05). We observed light monocytosis before treatment, which went down in count during the treatment period. In the normal condition, monocytes count in blood was in the range of 0.3–0.8 × 10^3^ cell/mm^3^.

In contrast, BMI of the TB patients rose gradually but significant (*P* = .012) after two (median 19.18 kg/m^2^) and the six-month of treatment. By the end of therapy, all patients' weight reached the highest BMI mean (median 20.36 kg/m^2^). (Figure 1(d)). There was a significant correlation (*P*-value .012 < .05) albeit weak (*r* = −0.281) between the decrease in suPAR with the increasing of BMI. 

Finally, thoracic imaging of the patients who were diagnosed to have active pulmonary TB showed far advanced lesions (*n* = 12), moderate advanced lesions (*n* = 7), and minimal lesions (*n* = 1). However, there was no significant correlation between suPAR level with the width of lesions on thoracic imaging of the TB patients before and after treatment (Table 3).

## 4. Discussion

TB has remained in the third rank of infectious disease in Indonesia for years. Lack of accurate method in monitoring progress in TB therapy could be one of the causes of this problem. The present study aimed to investigate the possible use of suPAR as a biomarker in pulmonary TB-AFB(+) patients, hence allow stratification of treatment regimens with possibly shortening of treatment in a majority of patients that may not require a six-month treatment period and intensified regimens in those with an increased risk for poor response and relapse.

It is surprising to see in general that the range of suPAR level in patients with active pulmonary TB (8.539 ng/mL–28.000 ng/mL) was rather different from African people which ranged between 0.9 ng/mL to 45 ng/mL [2]. Whether these are genetically influenced, it needs further study. It was found that suPAR level was elevated before treatment and drastically dropped after two- and four- month therapy. A decrease in suPAR after 8-month of Tb-treatment among treatment responders was also found in the study of Eugen-Olsen et al. [3]. Increased suPAR levels pretreatment may be a result of mobilization of macrophage into the bronchi and the increased immune activation and inflammation caused by the active infection. Adherence and migration of monocytes involves a functional interaction between uPAR and integrins [8]. The reduction trend of suPAR during six-month therapy was significantly in accordance with the decline in ESR, however, only significantly correlated with monocyte count. We only found light monocytosis before the treatment which decreased in count from time to time during anti-TB drugs treatment, the same is found in previous finding [9] that showed that mild absolute or relative monocytosis was found in 29% to 60% of TB patients. Considering that suPAR attach on monocytes and macrophage and play a role in cell migration [10], however, seems that in this study, the level of suPAR did not reflect the number of monocytosis. It is possible that release of suPAR in the blood not necessary statistically comparable with the process of monocytosis. 

The drastic decrease of suPAR level in the first two months was weakly associated with raise of BMI. The raise of appetite of TB patient might be due to decreasing of TNF-alpha release [11]. These acts affect the immune status condition which sifted to Th2, resulting in the pressure on Th1 or the existence of Th1 signal transduction resistance on macrophage by *tuberculosis* bacilli and infection severity [8]. 

By the end of the initial phase treatment, there were five patients who did not undergo sputum conversion yet, but at the end of continuation phase, all the patients underwent sputum conversion. Patient's sputum conversion occurred in 75%–85% cases after 2-month treatment and 96% cases after six-month treatment [9]. Although the trend of the drop of suPAR level and sputum conversion look similar, nevertheless it is hard to correlate the sputum conversion with the level of suPAR in the blood, since suPAR is not a direct product of *Mycobacterium*. 

Patients who have active pulmonary TB with AFB sputum(+) were found mostly in pulmonary TB patients with far advanced lesion, followed by moderate advanced lesion then minimal lesion. Pulmonary lesion is considered to be a key parameter used to diagnose and monitor the outcome of the treatment. As the disease progresses its resulted in the softening of caseosa necrosis, forming cavity containing a lot of *M. tuberculosis* and bronchial wall erosion which then are forced to come out through cough [12]. It is shown that in thoracic imaging can occur in one to three first month of anti-TB drugs treatment, and this can be better or stable in six months in 90% cases [9]. However, after 6 months of treatments there was one patient who still underwent destroyed pulmonary with wider* atelectasis*. In this study, suPAR level among patients with far advanced lesion, moderate advanced lesion, and minimal lesion did not differ significantly. This is in contrast with finding previous finding [13] in which the suPAR level correlated significantly with width of lesion. To our knowledge, these could be due to the use of different method in measuring the lesion. 

An interesting phenomenon was found in which the suPAR level was much higher (28.000 ng/mL) in one patient with pulmonary TB miliary type. Pulmonary TB miliary type is known as a severe type of pulmonary TB due to hematogen spread process that affect all parts of the lung [10]. Whether this one case reflects severity of the disease due to hematogen spread still needs further proof in further study.

This was the first research done in Indonesia studying the suPAR level during anti-TB drugs treatment. This research was aimed at obtaining the applicable use of suPAR as a complement to monitor treatment efficacy. All 20 patients in the current study responded clinically to the TB treatment. The positive effect of treatment was also reflected in decrease in the inflammatory status as measured by suPAR levels. Thus, we conclude that decreasing suPAR level may reflect treatment efficacy, but further studies involving TB patients that do not respond to TB treatment is necessary to determine if suPAR remains high for example, in TB-MDR patients. 

## 5. Conclusion

The decreasing of suPAR level in pulmonary TB patients with active AFB(+) was a manifestation of treatment efficacy. suPAR level elevated almost three times in TB patients before treatment and drastically dropped after the first two-month treatment, followed by the gradual decrease until the end of sixth month treatment that was comparable to suPAR level of the healthy control. The reduction trend of suPAR level was correlated to other clinical and laboratory parameters, that is, disappearance of patients' complaints, increasing of BMI, declining of ESR, and fewer monocytes number. However, suPAR concentration did not reflect the pulmonary lesion width on thoracic imaging; hence, suPAR could not be used as a sole biological marker to monitor the efficacy of the treatment.

## Figures and Tables

**Table 1:** Clinical data of patients included in the study.

No	Patients' characteristics	Frequency (n)	Percentage (%)
1	Number of patients	20	100%
	Male	10	50%
	Female	10	50%

2	Age (years); age median = 30 years		
	19–30	12	60%
	31–40	4	20%
	41–50	4	20%

3	Width of lesion classification		
	Minimal lesion	1	5%
	Moderate advance lesion	7	35%
	Far advance lesion	12	60%

4	Patients' complaints		
	Chronic cough >3 weeks	20	100%
	Decrease of appetite	15	75%
	Low grade fever	10	50%
	Shortness of breath	9	45%
	Hemoptoe	9	45%
	Chest pain	2	10%

**Table 2:** Comparison between medical records used to monitor TB theraphy progress in Indonesia, that is, patients complaints, BMI (kg/m^2^), width of lesion, sputum AFB, erythrocyte sedimentation rate (mm/hour), monocyte count (cell/mm^2^), and soluble urokinase plasminogen activator receptor during the 6-month therapy.

	During anti-TB-drugs treatment					
No.	Medical records and level of suPAR	Before Treatment	2 months	4 months	6 months	*P*-value <.05
1	Patients' Complaints					
	Chronic cough >3 weeks	20 (100%)	17 (85%)		5 (25%)	
	Decrease of appetite	15 (75%)	0 (0%)		0 (0%)	
	Low grade fever	10 (50%)	0 (0%)		0 (0%)	
	Shortness of breath	9 (45%)	5 (25%)		3 (15%)	
	Hemoptoe	9 (45%)	0 (0 %)		0(0 %)	
	Chest pain	2 (10 %)	1 (5 %)		1 (5 %)	

2	BMI	18.28 ± 2.12	19.18 ± 2.31		20.36 ± 2.35	.0001

3	Width of lesion classification		Thoracic Imaging *n* = 20		Thoracic Imaging *n* = 20	
	Minimal Lesion Mod Advance Lesion Far Advance Lesion	1 (5%) 7 (35%) 12 (60%)	$\left\{\begin{array}{c} \text{Worsen}:\;0\;(0\text{\%})\;\\ \text{Unchange}:\;3\;(15\text{\%})\\ \text{Getting}\;\text{better}:\;17\;(85\text{\%})\; \end{array}\right\}$		$\left\{\begin{array}{c} \text{Worsen}:\;1\;(5\text{\%})\\ \text{Unchange}:\;0\;(0\text{\%})\;\\ \text{Getting}\;\text{better}:\;19\;(95\text{\%}) \end{array}\right\}$	

4	Sputum AFB(+)	20 (100%)	5 (25%)		0(0%)	

5	ESR	84.15 ± 32.47	44.10 ± 29.97		19.30 ± 13.29	.0001

6	Monocytes Count	847.00 ± 244.52	662.00 ± 241.72		496.50 ± 129.67	0.0001

7	Level of suPAR	12.775 ± 3.940	8.019 ± 1.458	5.771 ± 0.796	5.009 ± 0.842	.0001

**Table 3:** Result of ANOVA Test.

No	Width of lesion classification	Test	Sig	Sig < 0.05	Conclusion
1	Min lession, mod advance, far advance	Anova test	Sig F	0.901	Not Significant
2	Min lession, mod advance	T-test	Sig t	0.61	Not Significant
3	Min lession, far advance	T-test	Sig t	0.792	Not Significant
4	Mod advance, far advance	T-test	Sig t	0.716	Not Significant
